# Supplementary material for: Population Dynamics between Erwinia amylovora, Pantoea agglomerans and Bacteriophages: Exploiting Synergy and Competition to Improve Phage Cocktail Efficacy
Source: Microorganisms. 2020 Sep 22;8(9):1449. doi: 10.3390/microorganisms8091449 (PMC7563384; doi:10.3390/microorganisms8091449)
Supplement: Supplementary file 1 [file microorganisms-08-01449-s001.pdf]

microorganisms

MDPI

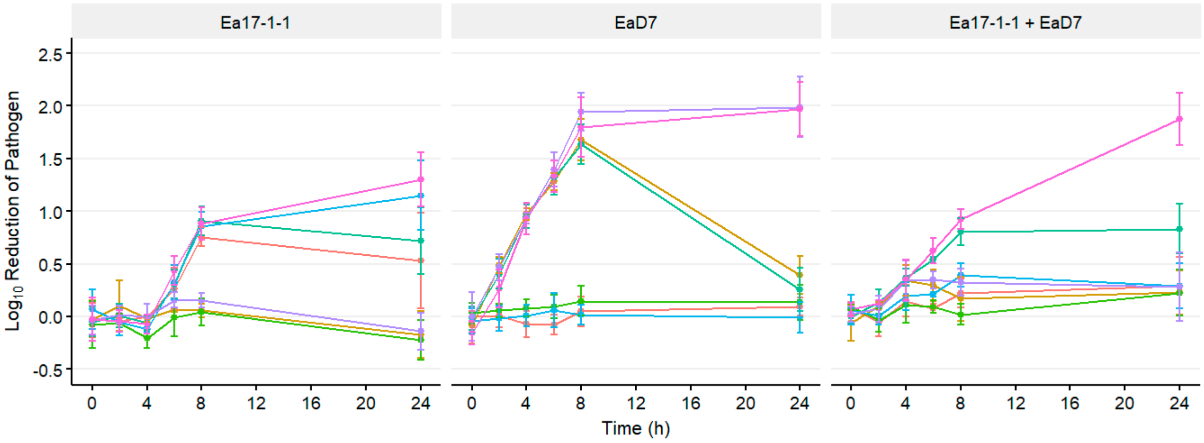

Figure S1. Log_{10} reduction of *E. amylovora* populations infected with different phage combinations compared to uninfected control. The infected strains Ea17-1-1, EaD7, and an equal combination of both (EaMix) are indicated in the top banners. Each host was infected by all possible phage combinations which are indicated by colour: ϕEa21-4 (●), ϕEa46-1-A1 (●), ϕEa35-70 (●), ϕEa21-4 + ϕEa46-1-A1 (●), ϕEa21-4 + ϕEa35-70 (●), ϕEa46-1-A1 + ϕEa35-70 (●), ϕEa21-4 + ϕEa46-1-A1 + ϕEa35-70 (●). Data are the mean ± SD of three replicates.

microorganisms

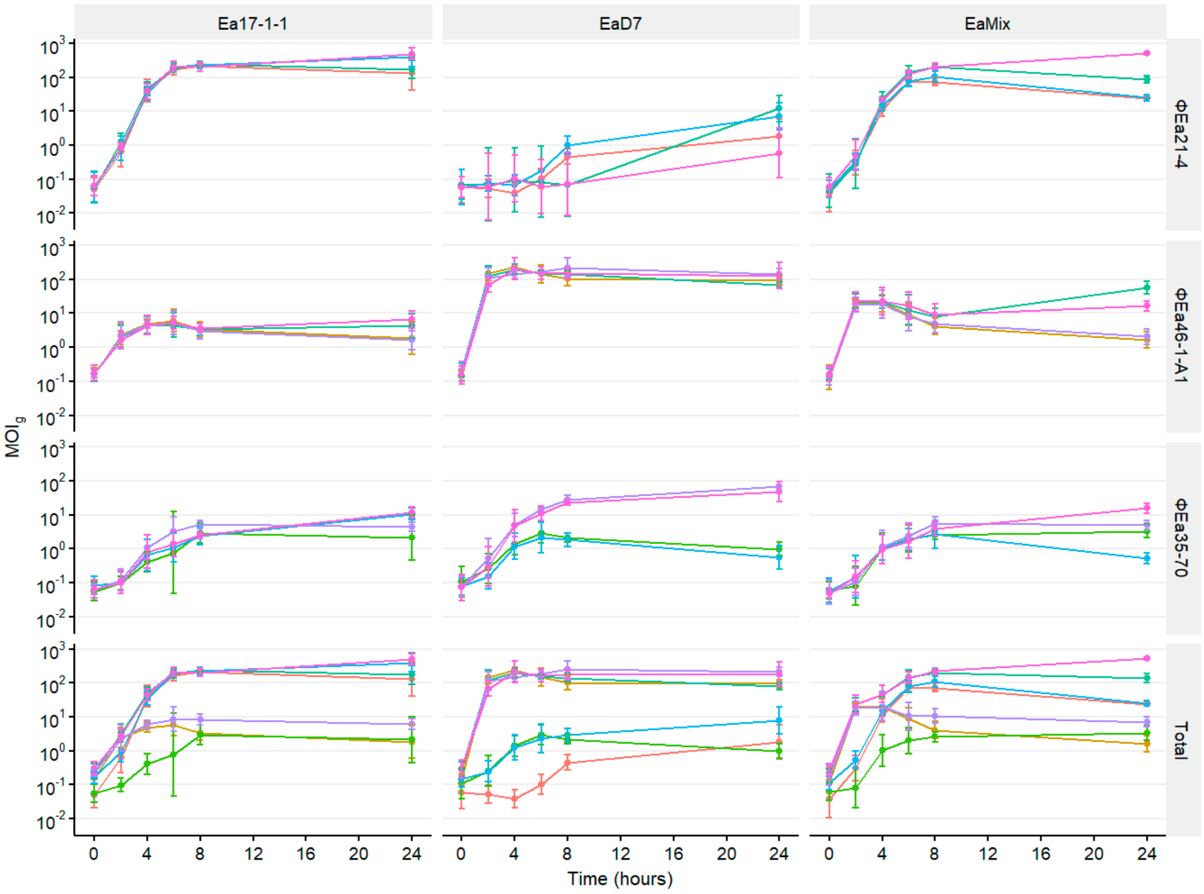

Figure S2. Ratio of phage genomes to *E. amylovora* genomes (MOI$_g$) over time in *E. amylovora* cultures infected with different phage combinations. The infected strains Ea17-1-1, EaD7, and an equal combination of both (EaMix) are indicated in the top banners. Each host was infected by all possible phage combinations which are indicated by colour: ϕEa21-4 (●), ϕEa46-1-A1 (●), ϕEa35-70 (●), ϕEa21-4 + ϕEa46-1-A1 (●), ϕEa21-4 + ϕEa35-70 (●), ϕEa46-1-A1 + ϕEa35-70 (●), ϕEa21-4 + ϕEa46-1-A1 + ϕEa35-70 (●). The MOI$_g$ was calculated for each phage individually and the sum of all phage genomes was also used to determine a total MOI$_g$, all of which are indicated in the banners on the right. Data are the mean ± SD of three replicates.

microorganisms

MDPI

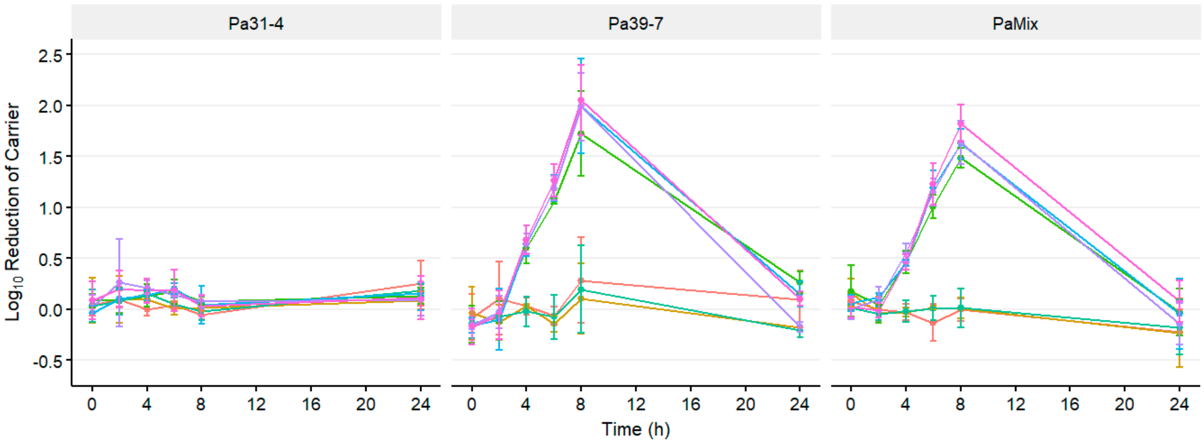

Figure S3. Log_{10} reduction of *P. agglomerans* populations infected with different phage combinations compared to uninfected control. The infected strains Pa31-4, Pa39-7, and an equal combination of both (PaMix) are indicated in the top banners. Each host was infected by all possible phage combinations which are indicated by colour: ϕEa21-4 (●), ϕEa46-1-A1 (●), ϕEa35-70 (●), ϕEa21-4 + ϕEa46-1-A1 (●), ϕEa21-4 + ϕEa35-70 (●), ϕEa46-1-A1 + ϕEa35-70 (●), ϕEa21-4 + ϕEa46-1-A1 + ϕEa35-70 (●). Data are the mean ± SD of three replicates.

microorganisms

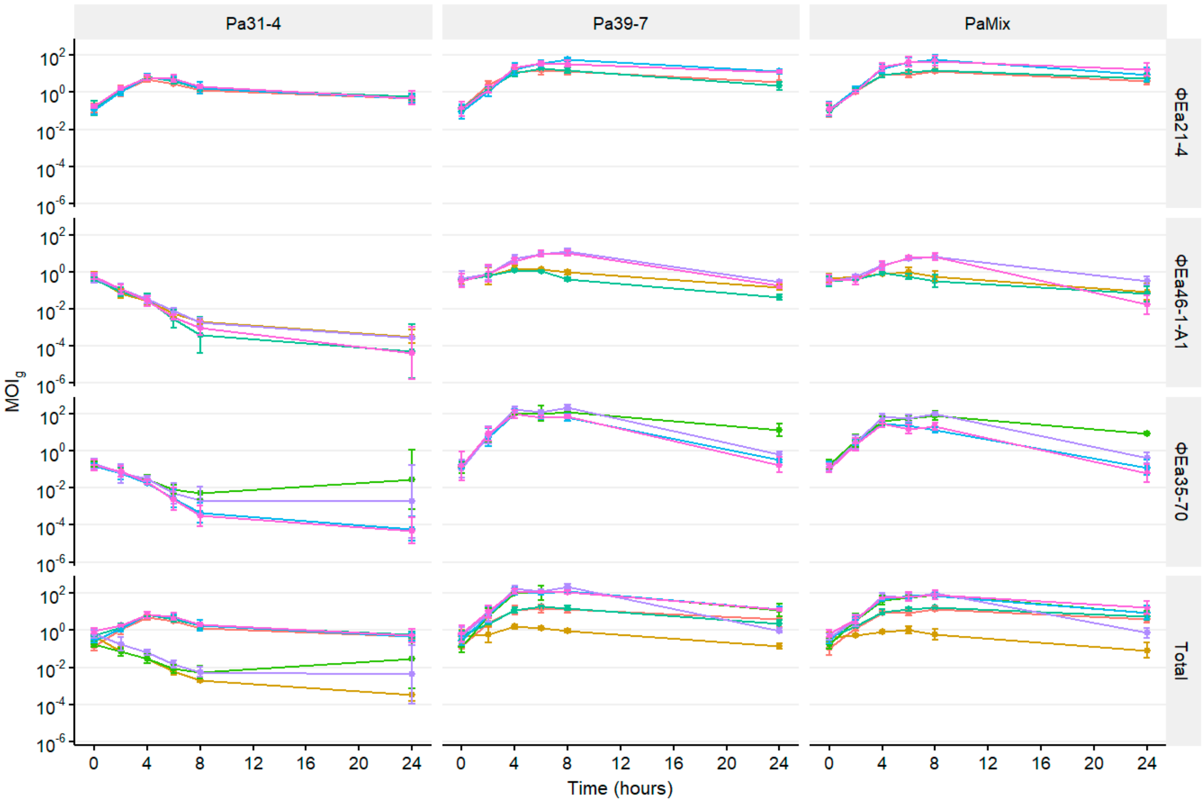

Figure S4. Ratio of phage genomes to *P. agglomerans* genomes (MOI_g) over time in *P. agglomerans* cultures infected with different phage combinations. The infected strains Pa31-4, Pa39-7, and an equal combination of both (PaMix) are indicated in the top banners. Each host was infected by all possible phage combinations which are indicated by colour: ϕEa21-4 (●), ϕEa46-1-A1 (●), ϕEa35-70 (●), ϕEa21-4 + ϕEa46-1-A1 (●), ϕEa21-4 + ϕEa35-70 (●), ϕEa46-1-A1 + ϕEa35-70 (●), ϕEa21-4 + ϕEa46-1-A1 + ϕEa35-70 (●). The MOI_g was calculated for each phage individually and the sum of all phage genomes was also used to determine a total MOI_g, all of which are indicated in the banners on the right. Data are the mean ± SD of three replicates.

microorganisms MDPI

Table S1. Primers and probes used for real-time qPCR.

Name	Species	Amplicon Size (bp)	Sequence (5'-3')
END37-F<br>END37-R<br>END37-P	*Erwinia virus Ea214*	149	TTCAGCTTTAGCGGCTTCGAGA<br>AGCAAGCCCTTGAGGTAATGGA<br>/56-ROXN/AGTCGGTACACCTGCAACGTCAAGAT/3IAbRQSp/
STS3-F<br>STS3-R<br>STS3-P	*Erwinia virus Era103*	96	GACAAACAAGAACGCGGCAACTGA<br>ATACCCAGCAAGGCGTCAACCTTA<br>/56-FAM/AGATGAAGTAGGTTATCTTCACAGTGCCCT/3BHQ_1/
N14-F<br>N14-R<br>N14-P	*Erwinia virus Ea9-2*	168	CATTGGGTAATCCCTTTGAG<br>GATAGACTGGTTCCCCTGTG<br>/56-FAM/TCTGGTGGA/ZEN/CAGAGACGATGTAAT/3IABkFQ/
RDH311-F<br>RDH311-R<br>RDH311-P	*Erwinia virus Ea35-70*	183	TGGAAGGTCTTCTTCGAGAC<br>GACTACCTGGGGATGTTCAG<br>/56-ROXN/GACGGAAAAGATCACGGTACTCTT/3IAbRQSp/
Ea-Lsc-F<br>Ea-Lsc-R<br>Ea-Lsc-P	*E. amylovora*	105	CGCTAACAGCAGATCGCA<br>AAATACGCGCACGACCAT<br>/5Cy5/CTGATAATCCGCAATTCCAGGATG/3IAbRQsp/
Pa-Gnd-F<br>Pa-Gnd-R<br>Pa-Gnd-P	*P. agglomerans*	73	TGGATGAAGCAGCGAACA<br>GACAGAGGTTCGCCGAGA<br>/5HEX/AAATGGACCAGCCAGAGCTCACTG/3BHQ_1/
